# A tiered approach to prioritizing registered pesticides for potential cancer hazard evaluations: implications for decision making

**DOI:** 10.1186/s12940-021-00696-0

**Published:** 2021-02-12

**Authors:** Pamela J. Schwingl, Ruth M. Lunn, Suril S. Mehta

**Affiliations:** 1 Integrated Laboratory Systems, Research Triangle Park, NC USA; 2 Office of the Report on Carcinogens, Division of the National Toxicology Program, National Institute of Environmental Health Sciences, P.O. Box 12233, Mail Drop K2-14, Research Triangle Park, NC 27709 USA

**Keywords:** Hazard identification, Pesticides, Cancer, Epidemiology, Scoping, Evidence mapping

## Abstract

**Background:**

Over 800 pesticides are registered for use in the United States. Human studies indicate concern that some pesticides currently in use in large quantities may also pose a carcinogenic hazard. Our objective is to identify candidates for future hazard evaluations among pesticides used in high volumes in the United States and also classified as potential carcinogens by U.S. Environmental Protection Agency (USEPA). We also identify data gaps where further research is needed.

**Methods:**

We used a systematic, two-tiered review approach to prioritize pesticides. First, we identified currently registered pesticides classified by USEPA as “possible”, “suggestive”, or “likely” human carcinogens. Among these, we selected pesticides USEPA has listed as commonly used by volume in at least one sector (agriculture, home and garden, or industry, commercial, and/or government), and those without a published hazard evaluation in the past 5 years. Second, we searched primary literature databases for peer-reviewed human cancer studies reporting pesticide-specific data published since the last USEPA carcinogenicity evaluation for each pesticide, and created evidence maps of the number of studies meeting our criteria for each identified pesticide. No evaluation of study results or risk-of-bias assessments were conducted.

**Results:**

We identified 18 pesticides meeting our selection criteria, 16 pesticides had information from human cancer studies published after their initial carcinogenicity review. Of these, eight pesticides had at least three studies for one or more cancer sites: carbaryl, dichloropropene, dimethoate, mancozeb, metolachlor, pendimethalin, permethrin, and trifluralin. A major limitation in the literature revealed a shortage of studies reporting risk estimates for individual pesticides, rather pesticides were grouped by chemical class.

**Conclusions:**

Our scoping report provides a map of the existing literature on real-world exposures and human cancer that has accumulated on pesticides classified as potential carcinogens by USEPA and used in high volumes. We also illustrate that several pesticides which are “data-rich” may warrant updated authoritative hazard evaluations. Our two-tiered approach and utilization of evidence mapping can be used to inform future decision-making to update cancer hazard evaluations.

**Supplementary Information:**

The online version contains supplementary material available at 10.1186/s12940-021-00696-0.

## Background

Although pesticides have many benefits, they are also associated with adverse health outcomes including cancer, which is especially concerning given their pervasive use. A hazard assessment is an initial and necessary step to raise the awareness of pesticide hazards to the public and to those most likely to be exposed, laying the groundwork for a formal risk assessment. In the United States, these assessments can provide Federal, state, and local health regulatory and research agencies with critical information needed to identify emerging public health issues, conduct formal risk assessments, and focus research where it is needed most, and if warranted, further restrict or ban use. We discuss U.S. pesticide exposures, health outcomes, regulatory concerns, and the need to prioritize pesticides for hazard assessments.

### Exposure concerns

Conventional pesticides, including insecticides, fungicides, herbicides, plant growth regulators, rodenticides, and other compounds, are widely used in the U.S., and their use has changed considerably over the past 50 years. In 1960, 196 million pounds of pesticides were applied to crops [1]. By 2012, total U.S. pesticide use had increased to more than 1 billion pounds [2]. Not only does the increase in use potentially impact those occupationally involved in the production and application of pesticides, but also the general population via inhalation, ingestion, dermal contact, and/or ocular contact from consumer products, food, crops, foliage, or soils containing pesticides [3, 4]. While approximately 66% of pesticide expenditures in 2012 were in the agricultural sector, the home and garden sector constituted 24% of pesticide expenditures, directly or indirectly exposing the public to residues in outdoor recreational spaces and gardens, as well as in homes, schools, and offices [2, 5, 6]. In the U.S., more than 90% of the population are estimated to have detectable concentrations of pesticide biomarkers in their urine or blood [7]. For any individual, the level and impact of exposure will vary depending on age, occupation, home residence near pesticide applications, treatment of a residence with pesticides, involvement in a pesticide spill, the nature and volatility of the compound, or the persistence of the pesticide itself [8], as well as socioeconomic status, race/ethnicity, and underlying health conditions.

### Health concerns

The pervasive use of pesticides offers benefits to public health, not the least of which is increased crop and livestock protection, and control of disease vectors [9]. Given the widespread destruction of crops by pests, herbicide and fungicide use beginning in the 1970’s has increased agricultural yield of quality foods at affordable prices [10]. Since 2000, progress in malaria control resulted in a 60% decrease in mortality and millions of cases averted, primarily due to expanded access to vector-control insecticide-based interventions (e.g., long-lasting insecticidal bed nets and indoor residual spraying) [11].

However, pesticides have also increased the burden of health effects in humans, including among others, dermatological, gastrointestinal, neurological, carcinogenic, respiratory, reproductive, and endocrine effects [12]. Most bioactive chemicals in pesticides are inherently toxic and it is well accepted that acute poisonings cause health effects such as seizures, rashes, and gastrointestinal illness [13, 14]. Pesticides are designed in specific ways to attack insects and plants. For example, cholinesterase inhibitors (e.g., organophosphate [OP] and carbamate insecticides) interfere with nerve impulse transmission at the synapse gap. Pyrethroid and neonicotinoid insecticides are synthetic chemicals based on the molecular structure of naturally occurring compounds and act on the insect’s nervous system. Insect growth regulators are chemicals based on hormones that regulate arthropod development and disrupt metamorphosis during immature stages [15–17]. Due to the similarity of many of these processes to those in human biochemistry, it is possible that pesticides pose a threat to the health of humans depending on the nature of the chemical and the level of exposure.

The toxicity of pesticides also poses the potential for increased cancer risk [8]. The International Agency for Research on Cancer (IARC) [18] and the U.S. National Toxicology Program’s (NTP) Report on Carcinogens (RoC) [19] have classified many pesticides as known or suspected human carcinogens. A growing body of epidemiologic and molecular studies associate pesticides used in agricultural, commercial, and home and garden applications with excess cancer risk. Specific cancers include prostate cancer, non-Hodgkin lymphoma (NHL), leukemia, childhood leukemia, multiple myeloma, and breast cancer [20–22]. Some of these cancers have been increasing at least since 2007. For instance, the overall cancer incidence rate in children (0–14 years of age) has been increasing by 0.8% per year; acute lymphocytic leukemia (ALL) has increased 1% annually, and chronic myeloid leukemia (CML) and acute myeloid leukemia (CML) have increased 2% annually; and breast cancer incidence has increased 0.3% annually [23].

### Hazard prioritization and risk assessment

The U.S. Environmental Protection Agency (USEPA) is mandated to regulate pesticides to prevent unreasonable adverse effects on human health or the environment (*The Federal Insecticide, Fungicide, and Rodenticide Act (FIFRA)* [24]*),* and to establish maximum permissible levels for pesticide residues in food (*The Federal Food, Drug, and Cosmetic Act (FFDCA)* [25]). These laws also mandate USEPA to conduct a re-registration and hazard evaluation of registered pesticides within 15 years of initial registration and use data published since initial registration to update carcinogenicity classifications. In the past, USEPA has revoked, cancelled, or allowed registrations to expire, effectively “banning” their use in the U.S. (e.g., lindane, dichlorodiphenyltrichloroethane (DDT), arsenic trioxide, sodium arsenate, heptachlor). USEPA has also used the “restricted use product status” to manage the use of potentially carcinogenic pesticides (e.g., pentachlorophenol) to limit what crops the pesticide can be used on, specifying safety equipment to be worn by applicators, setbacks from sensitive habitats, preharvest intervals, field re-entry intervals, or management practices that should be used to minimize off-target movement or drift [26].

Given the pervasiveness, increased use, potential carcinogenicity of certain pesticides, and growing database of epidemiologic studies, it is important to determine whether updated cancer hazard evaluations may be warranted and, if so, which pesticides should be prioritized.

For these reasons, we aim to systematically identify candidate pesticides for new cancer hazard or risk assessments by scoping the literature for recently available epidemiologic cancer studies of pesticides classified by USEPA as potential carcinogens and used in high volumes. Cancer epidemiology studies provide valuable information on the potential for specific types of cancer from post-market human exposure.

Currently there is no standardized approach to prioritizing chemicals for a hazard assessment. At minimum, the identification of a robust database of studies conducted in independent populations for each cancer site is required for a hazard evaluation, thus scoping the literature is a crucial first step in prioritizing chemicals for further evaluation [27, 28].

## Methods

We used a systematic, tiered two-part process to identify candidate pesticides for new cancer hazard assessments: (1) identification of high-volume registered pesticides with carcinogenic potential, and (2) literature searching, screening, and evidence mapping of cancer epidemiology studies of select pesticides.

### Part 1: identification of high-volume registered pesticides with carcinogenic potential

#### Identifying pesticides with carcinogenic potential

First, we searched the most recent *USEPA Pesticides Chemical Search database* (https://iaspub.epa.gov/apex/pesticides/f?p=chemicalsearch:1) for pesticides that were registered (i.e., currently registered, in re-registration, or pending registration). We excluded chemicals that were antimicrobials and biopesticides, and excluded any pesticides that were deregistered, banned, or determined by USEPA to be ineligible for re-registration.

To determine USEPA carcinogenicity ranking of pesticides (e.g., probable, possible, likely, suggestive of carcinogenicity), we used the most recent USEPA Office of Pesticide Program’s (OPP) list of pesticide chemicals evaluated for carcinogenic potential (*Chemicals Evaluated for Carcinogenic Potential Annual Cancer Report* [29]). In evaluating and describing the potential carcinogenicity of a pesticide, USEPA’s OPP follows USEPA *Guidelines for Carcinogen Risk Assessment* which describes the criteria used to arrive at a carcinogenicity classification; however, it should be noted that USEPA’s ranking of carcinogenicity “represents only the potential carcinogenicity hazard for the chemical with no consideration of exposure information … and is not intended to be used independent of the full risk assessment for the chemical” [30]. It should be noted that these evaluations are done primarily on parent chemicals, and not on commonly-used end use formulations.

The evaluation of pesticides began in 1986 and since then, USEPA has used a variety of terms to classify the carcinogenicity of pesticides ([29]; also see Supplemental Table S1). We included potentially carcinogenic pesticides by selecting those classified at the time of their registration as probable, possible, likely, or suggestive of being carcinogenic to humans (Group B, C) and excluded any pesticide not classifiable as a human carcinogen because of inadequate evidence or having evidence of non-carcinogenicity in humans (Group D, E).

#### Prioritization of “high- volume” pesticides

Next, we matched the registered pesticides having carcinogenic potential to a listing of pesticides most commonly used in the U.S. [2]. Though not directly indicative of U.S. human exposure, high-volume usage patterns can be used as an indirect proxy to examine the potential for occupational, agricultural, environmental, and residential exposure. USEPA provides estimates in millions of pounds for commonly used conventional pesticide active ingredients in 2012. These include the top 25 pesticide ingredients used in the agricultural market sector, the top 10 pesticide ingredients used in the home and garden market sector, and top 10 pesticide ingredients used in the industry/commercial/government market sector. In addition, estimates in millions of pounds for the 10 most commonly used OP insecticides in 2012 were reported.

We did not include any pesticide re-evaluated for carcinogenicity by IARC [18] and/or RoC [19] within the past five years, as these pesticides have had a recent hazard evaluation.

### Part 2: literature searching, screening and evidence mapping of cancer epidemiology studies of selected pesticides

Our goal was to assess whether there is an adequate database to warrant a cancer hazard evaluation, thus we did not evaluate the results of studies, but only extracted information about the number of studies providing estimates of risk for pesticides and the number of various cancer sites. In addition, no risk-of-bias assessments were conducted.

Given that cancer epidemiology studies rarely include specific information in the title or abstract for all chemicals analyzed, we used a more sensitive and comprehensive approach to searching than using the traditional title, abstract, or keyword searches. First, we searched citation databases, including PubMed, Web of Science, and Scopus through April 24, 2020 for epidemiology studies evaluating pesticides, herbicides and fungicides in relation to cancer using search strings described in Appendix. A total of 5494 citations were identified based on our search criteria; available .pdfs for these citations were automatically downloaded (*N* = 3620) and sorted into pesticide-specific mini-libraries. EndNote version X9 was used to conduct full-text searches for the pesticides identified in *Part 1*; .pdfs which listed the pesticide were screened to assess the presence of original cancer risk estimates for the pesticide or pesticide group. Those with original data were tagged by pesticide and cancer type in the Health Assessment Workspace Collaborative (HAWC) tool [31].

Studies referring only generally to pesticide exposure without any specific mention of one of the pesticides of interest were not included (e.g., occupation in agriculture without mention of specific pesticides). Those studies reporting individual risk estimates for the specific pesticide of interest were counted and are referred to as pesticide-specific studies. In addition, we counted studies which reported a grouped estimate of risk which included both the specific pesticide as well as others and refer to these as non-specific pesticide studies). For example, in studies reporting non-specific risk estimates the effect may be reported for a pesticide class (e.g., OPs), by carcinogenic potential as defined by authors using IARC categories (e.g., probable or possible carcinogens), by crops typically treated with the pesticide of interest (e.g., corn, wheat), or by type of residential or home use (e.g., termites, lawns, hair lice treatments). For example, risk estimates may be reported for a group of OPs that specifically included acephate; or the pesticide may be one of several pesticides mentioned and known to be used in a particular area or on particular crops and estimates are only crop- or location-specific.

As we were concerned with finding literature published after the last carcinogenicity review of the pesticide, we counted the number of studies published after each pesticide’s respective date listed in the USEPA 2017 report [29]; separately, studies published prior to the evaluation date were noted. When multiple reports of a pesticide-cancer association were published based on the same cohort over time, generally only the most recent study was counted. As such, the study count in this analysis mostly reflects the number of unique populations identified. However, for some populations, we identified multiple research articles from the same study population that examined different facets of the particular pesticide-cancer relationship. Based on these results, an evidence map by cancer site was created in HAWC and we visualized the results using Tableau version 10.5.

## Results

### Part 1: identification of high-volume registered pesticides with carcinogenic potential

**Fig. 1:**
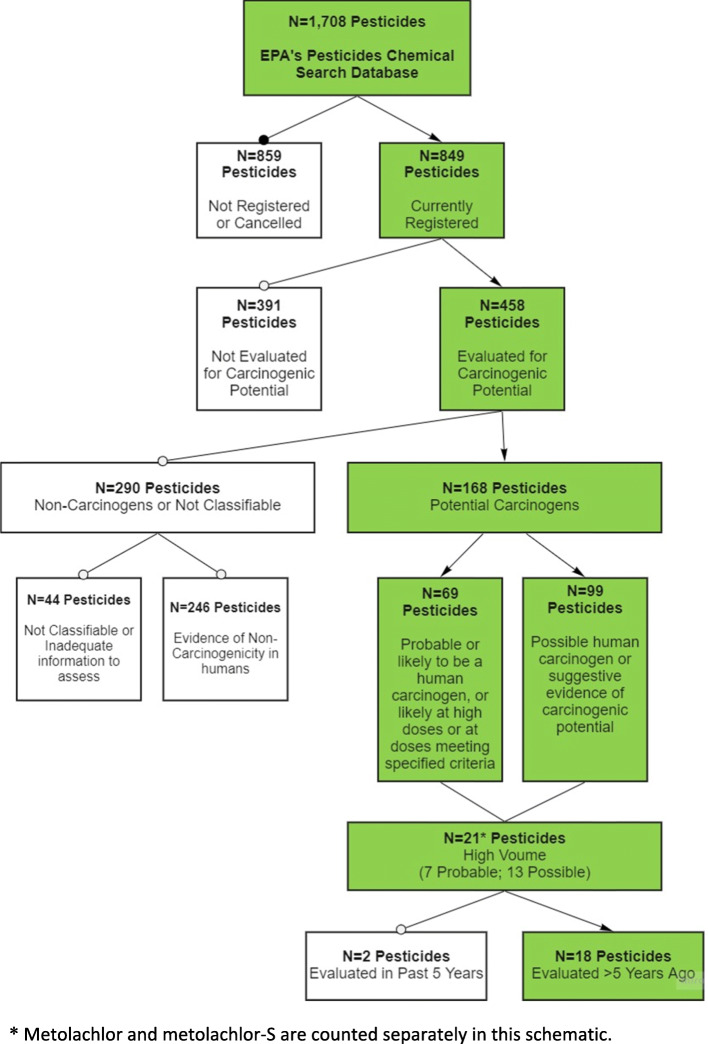
Flow chart illustrating selection of pesticides for scoping review. * Metolachlor and metolachlor-S are counted separately in this schematic

We identified 18 currently registered pesticides classified as probable or possible human carcinogens by USEPA, used in high volumes in the U.S., and with no recent hazard evaluation conducted within the past 5 years by IARC or RoC as eligible for literature scoping (Fig. 1; Table 1). Briefly, of the 1708 pesticides included in USEPA’s pesticides database, 849 are currently listed as registered for use. The remaining pesticides (*N* = 859) have no process associated with them, indicating they are not currently registered for use in U.S. or their use has been cancelled. Of the 849 pesticides registered for use, 526 were listed by USEPA as having been evaluated for cancer hazard potential (62.0%). Of these, we excluded those not currently registered by USEPA (*N* = 68), leaving 458 registered pesticides evaluated for carcinogenic potential.

**Table 1:**
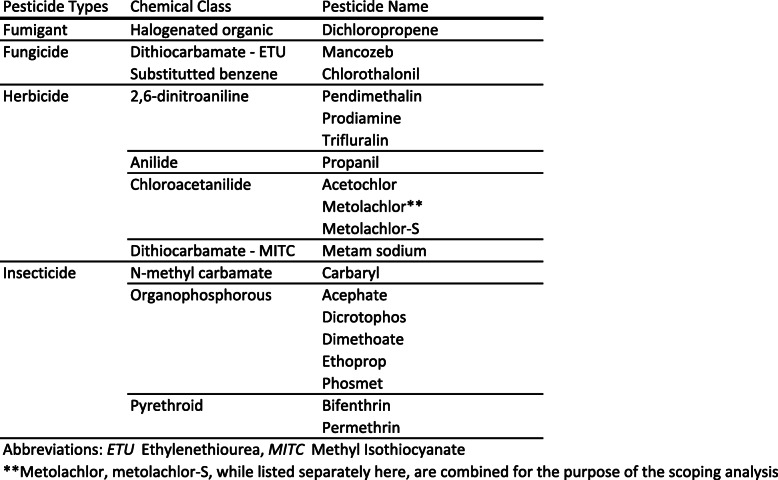
Eighteen pesticides meeting scoping criteria by pesticide type, pesticide class, and pesticide name

Abbreviations: *ETU* Ethylenethiourea, *MITC* Methyl Isothiocyanate

**Metolachlor, metolachlor-S, while listed separately here, are combined for the purpose of the scoping analysis

Among these 458 registered pesticides evaluated for carcinogenicity, 69 were classified by USEPA as either probable or likely to be human carcinogens and 99 were classified as possible human carcinogens, totaling 168 potential carcinogens. The remaining 290 pesticides were excluded as USEPA reported that they were either “not likely to be carcinogenic to humans” or “(having) evidence of non-carcinogenicity in humans” (*N* = 246), or “not classifiable as a human carcinogen” or had “inadequate data for an assessment of human carcinogenic potential” (*N* = 44).

Of the 168 pesticides classified as potential carcinogens, 21 were used in high volumes, including acephate, acetochlor, bifenthrin, carbaryl, chlorothalonil, dichloropropene, dicrotophos, dimethoate, ethoprop, mancozeb, metam sodium, metolachlor, metolachlor-S, pendimethalin, permethrin, phosmet, propanil, prodiamine, trifluralin, malathion, and tetrachlorvinphos. Metolachlor and metolachlor-S are listed separately in USEPA documents and are used in different products; however, they have the same chemical formula. As these two pesticides are typically not distinguished in the epidemiologic literature, and for the purposes of the literature scoping, they are counted together as one pesticide, leaving 20 pesticides for literature scoping. As the concern was with those pesticides without a recent hazard evaluation, malathion and tetrachlorvinphos were excluded because IARC evaluated them within the past 5 years.

**Fig. 2:**
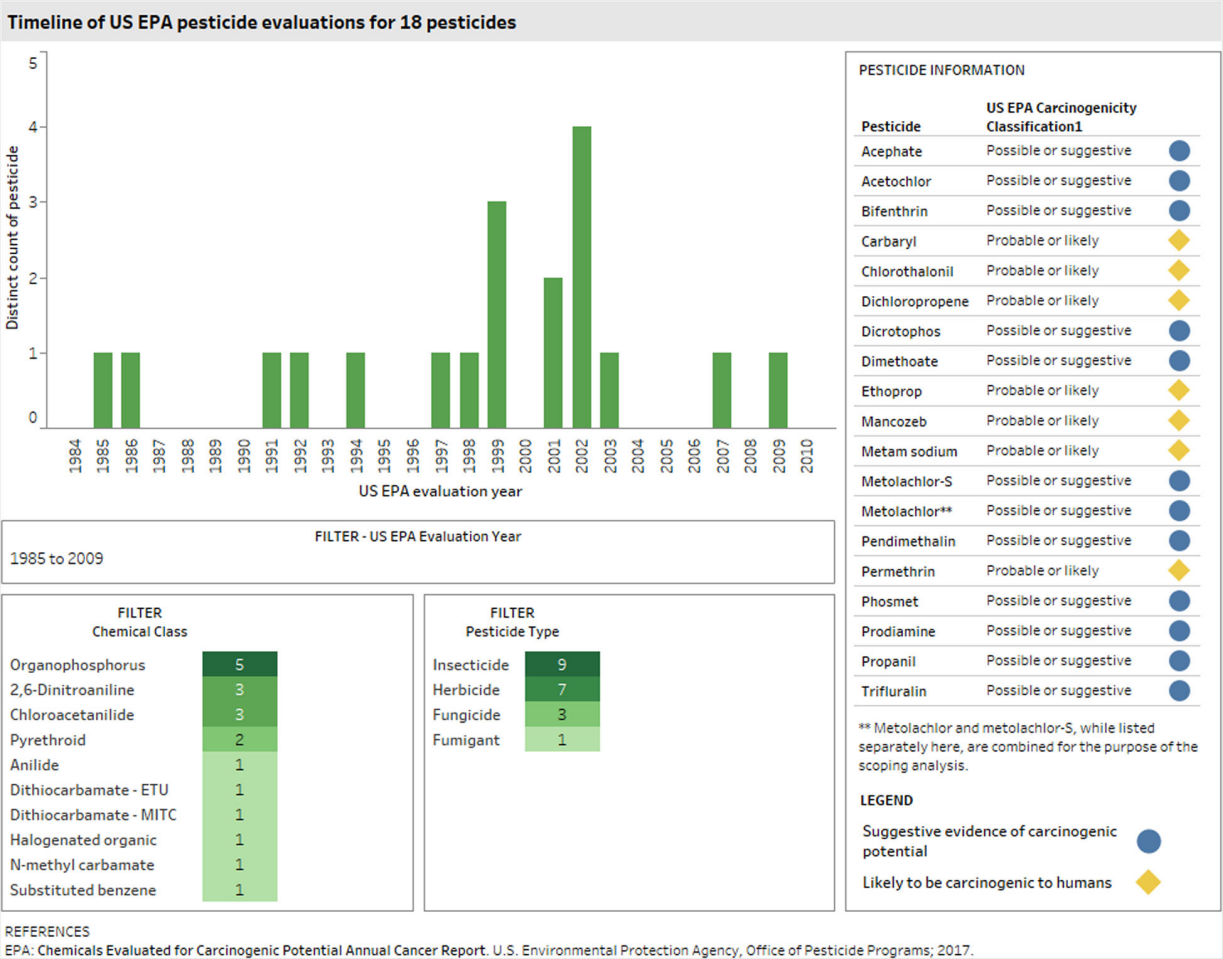
Timeline of USEPA pesticide evaluations for 18 pesticides

Among the 18 remaining pesticides, four have been evaluated for carcinogenicity by RoC and/or IARC more than 5 years ago (i.e., chlorothalonil, dichloropropene, permethrin, and trifluralin); the 14 other pesticides have not been evaluated by IARC or RoC. The 18 high-volume pesticides were evaluated by USEPA for carcinogenicity during the years 1985 through 2009, with approximately half being initially evaluated more than 20 years ago (Tableau Dashboard (Fig. 2; https://public.tableau.com/profile/ntp.visuals#!/vizhome/ORoCPesticidesCancer081220/ReadMe).

Information about the volume of pesticide used across sectors for the 18 pesticides is based on USEPA’s 2008–2012 market estimate report (4) (Table 2; https://public.tableau.com/profile/ntp.visuals#!/vizhome/ORoCPesticidesCancer081220/ReadMe). Metolachlor-S, dichloropropene, metam sodium, acetochlor, chlorothalonil, pendimethalin, acephate, mancozeb, metolachlor, propanil, and trifluralin are among the top 25 highest use pesticides used in the agricultural market sector in 2012. Chlorothalonil, pendimethalin, acephate, bifenthrin, and prodiamine are listed among the top 10 pesticides used in the industry/commercial/government market sector. Pendimethalin, acephate, carbaryl, and permethrin are listed among the top 10 pesticides used in the home and garden market sector. Additionally, dicrotophos, dimethoate, ethoprop, and phosmet ranked among the top 10 OPs in 2012.

**Table 2:**
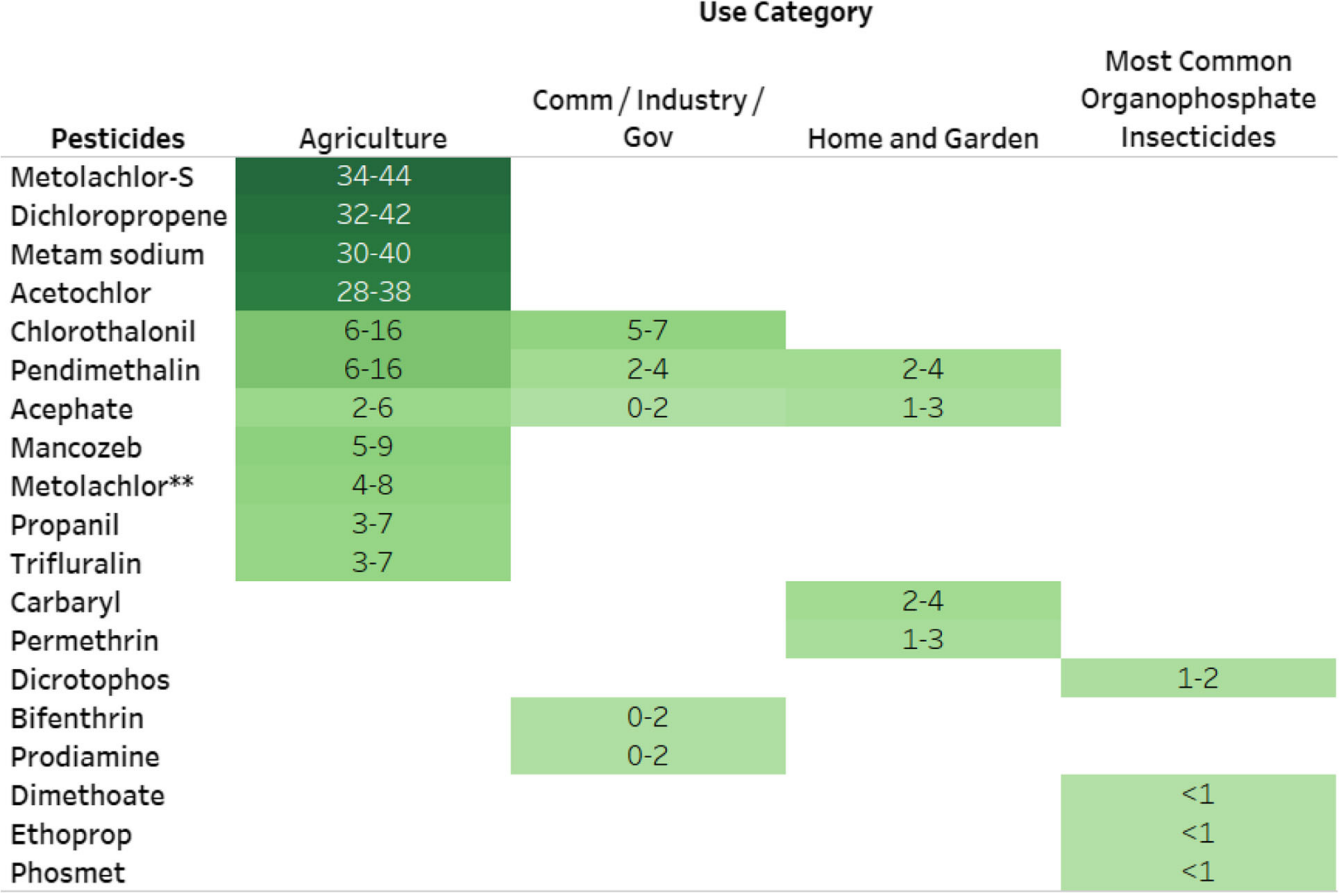
Pounds of use (in millions) of 18 registered pesticides with carcinogenic potential used in high volumes

Abbreviations: *Comm/Industry/Gov* Commercial / Industry/ Governmental sector

** Metolachlor, metolachlor-S, while listed separately here, are combined for the purpose of the scoping analysis

### Part 2: literature searching, screening and evidence mapping of cancer epidemiology studies of selected pesticides

An interactive Tableau dashboard shows all details of the results of the literature screening (Evidence Map https://public.tableau.com/profile/ntp.visuals#!/vizhome/ORoCPesticidesCancer081220/ReadMe). Sixty-six (*N* = 66) unique publications were identified that report pesticide-specific data for 16 of the pesticides in relation to cancer; no publications were identified for cancer and prodiamine or dichrotophos (Table 3; https://public.tableau.com/profile/ntp.visuals#!/vizhome/ORoCPesticidesCancer081220/ReadMe). Data in these publications were based on 27 unique study populations, with two or more reports from 8 study populations, 28 reports from the Agricultural Health Study cohort, and one publication for each of the remaining 18 populations. About half (*N* = 34) of the publications were cohort studies, and slightly less than a half (*N* = 29) were case-control studies. A reference list of cancer epidemiology studies can be found in Supplemental Table S2.

**Table 3:**
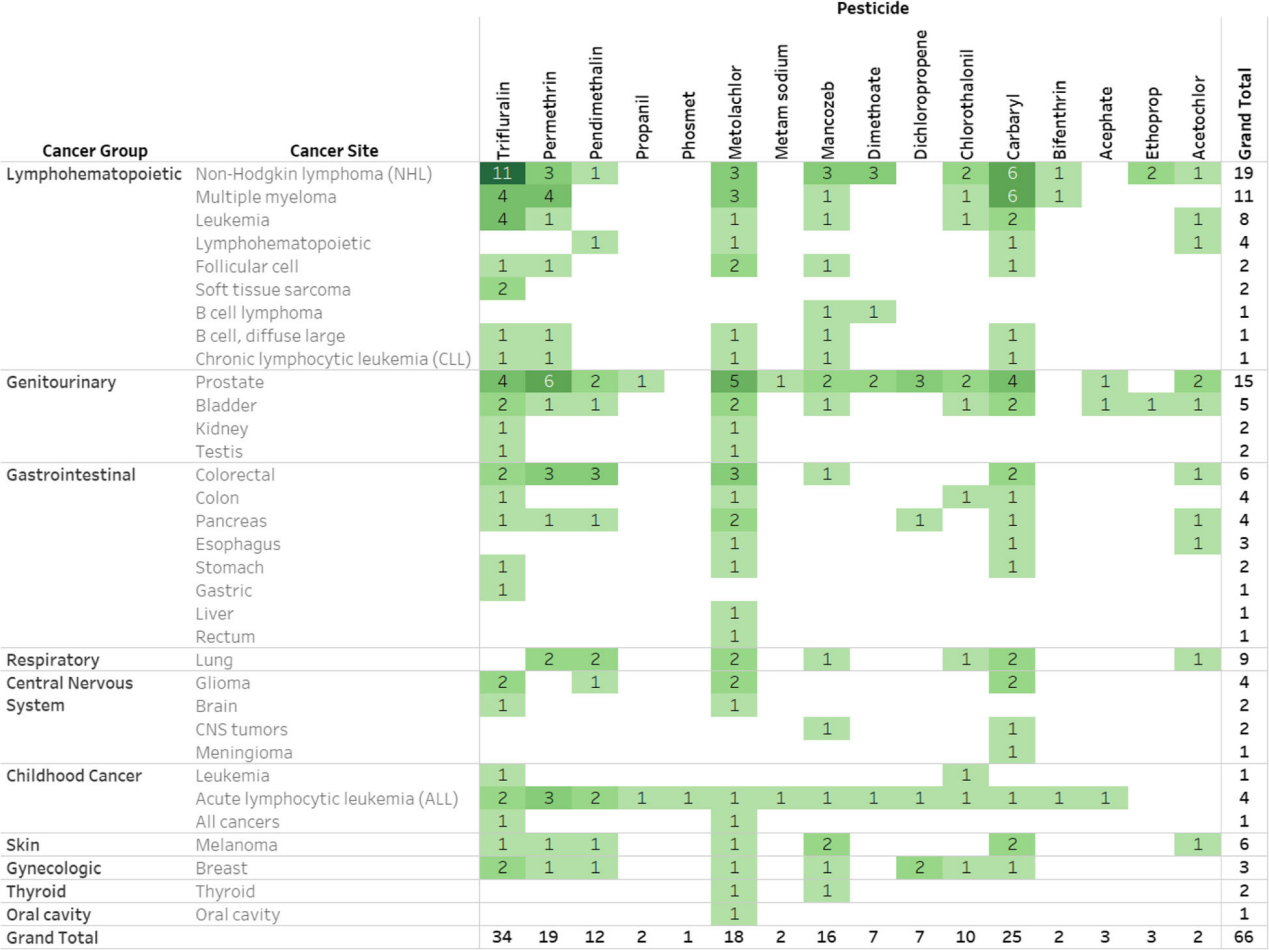
Frequency of cancer epidemiology studies with specific exposure estimates for 16 pesticides by cancer site

Studies for each cell shown online at https://public.tableau.com/profile/ntp.visuals#!/vizhome/ORoCPesticidesCancer081220/ReadMe

The most frequent cancer types associated with these pesticides were NHL (*N* = 19), prostate cancer (*N* = 15), multiple myeloma (*N* = 11), lung cancer (*N* = 9), and leukemia (*N* = 8). Multiple studies included results for multiple pesticides and multiple cancer sites; for example, any particular study might report risk estimates for carbaryl, dimethoate, and permethrin, each in relation to five or more cancer sites. Thus, the total number of reported estimates in the margins of the evidence map may exceed the total number of citations.

Eight of these pesticides are considered “data-rich”; that is, each pesticide has at least three reports from separate study populations for one or more cancer sites: carbaryl, dichloropropene, dimethoate, mancozeb, metolachlor, pendimethalin, permethrin, and trifluralin (Table 4; https://public.tableau.com/profile/ntp.visuals#!/vizhome/ORoCPesticidesCancer081220/ReadMe). The cancers most frequently reported on for these pesticides include NHL, multiple myeloma, leukemia, prostate cancer, colorectal cancer, and childhood ALL. Four of these pesticides (trifluralin, carbaryl, permethrin, and metolachlor) have five or more reports for at least one cancer site. Among the data-rich pesticides, there is an absence of pesticide-specific data available across cancer types for mancozeb, dimethoate, pendimethalin, and dichloropropene. In addition, pesticide-specific data are not available for leukemia, childhood ALL, and colorectal cancers for trifluralin, carbaryl, and metolachlor.

**Table 4:**
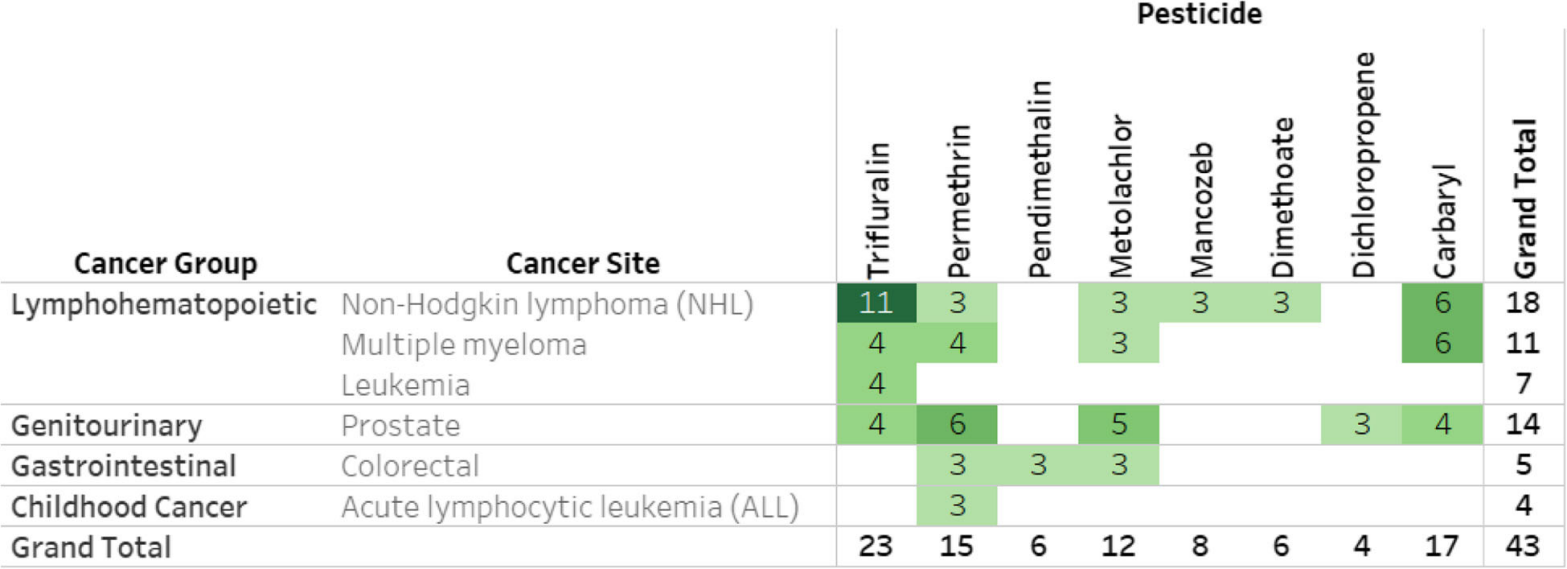
Frequency of cancer epidemiology studies with specific exposure estimates for eight “data-rich” pesticides by cancer site

Studies for each cell shown online at https://public.tableau.com/profile/ntp.visuals#!/vizhome/ORoCPesticidesCancer081220/ReadMe

Forty-seven studies (*N* = 47) of the 16 pesticide evaluations report non-specific risk estimates (Table 5); https://public.tableau.com/profile/ntp.visuals#!/vizhome/ORoCPesticidesCancer081220/ReadMe). Chlorothalonil and bifenthrin would also be considered “data-rich” when studies with specific and non-specific risk estimates are combined. The non-specific pesticide studies of NHL, leukemia, multiple myeloma, and prostate cancer in relation to mancozeb, permethrin, and trifluralin could add supplemental information to an already rich dataset. Furthermore, there are several studies with non-specific risk estimates for mancozeb, carbaryl, metolachlor and chlorothalonil in relation to breast cancer. However, many of the studies with non-specific risk estimates use ecologic study designs, and the lack of individual-level pesticide use data and the low-quality of exposure assessments may substantially weaken their utility in a hazard evaluation.

**Table 5:**
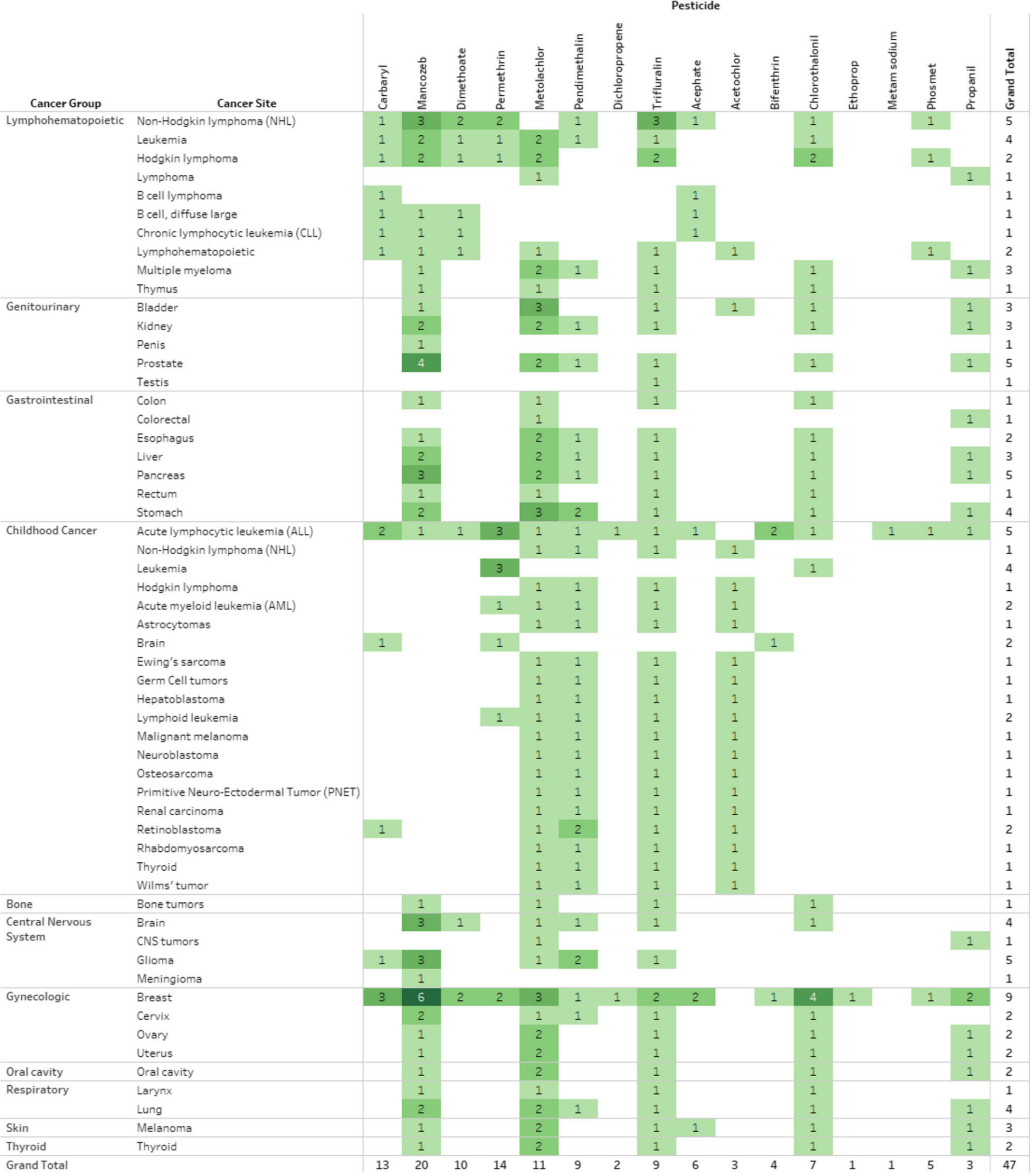
Frequency of cancer epidemiology studies for 16 pesticides with non-specific pesticide group risk estimates by cancer site

Studies for each cell shown online at https://public.tableau.com/profile/ntp.visuals#!/vizhome/ORoCPesticidesCancer081220/ReadMe

Our search also identified pesticide exposure and cancer studies published prior to their respective USEPA evaluations. While human studies published prior to the initial USEPA evaluations may have been considered at that time, we wanted to report on the total number of studies available for any updated hazard evaluation. Nine additional pre-USEPA evaluation reports providing pesticide-specific estimates are available, and 13 with non-specific risk estimates. Seven of the pesticide specific estimates are for carbaryl (Table 6). When the USEPA pre-evaluation studies for carbaryl are counted, there are nine pesticide-specific studies for NHL, seven for multiple myeloma, four for prostate cancer, and three each for leukemia and lung cancer (Table 6; https://public.tableau.com/profile/ntp.visuals#!/vizhome/ORoCPesticidesCancer081220/ReadMe).

**Table 6:**
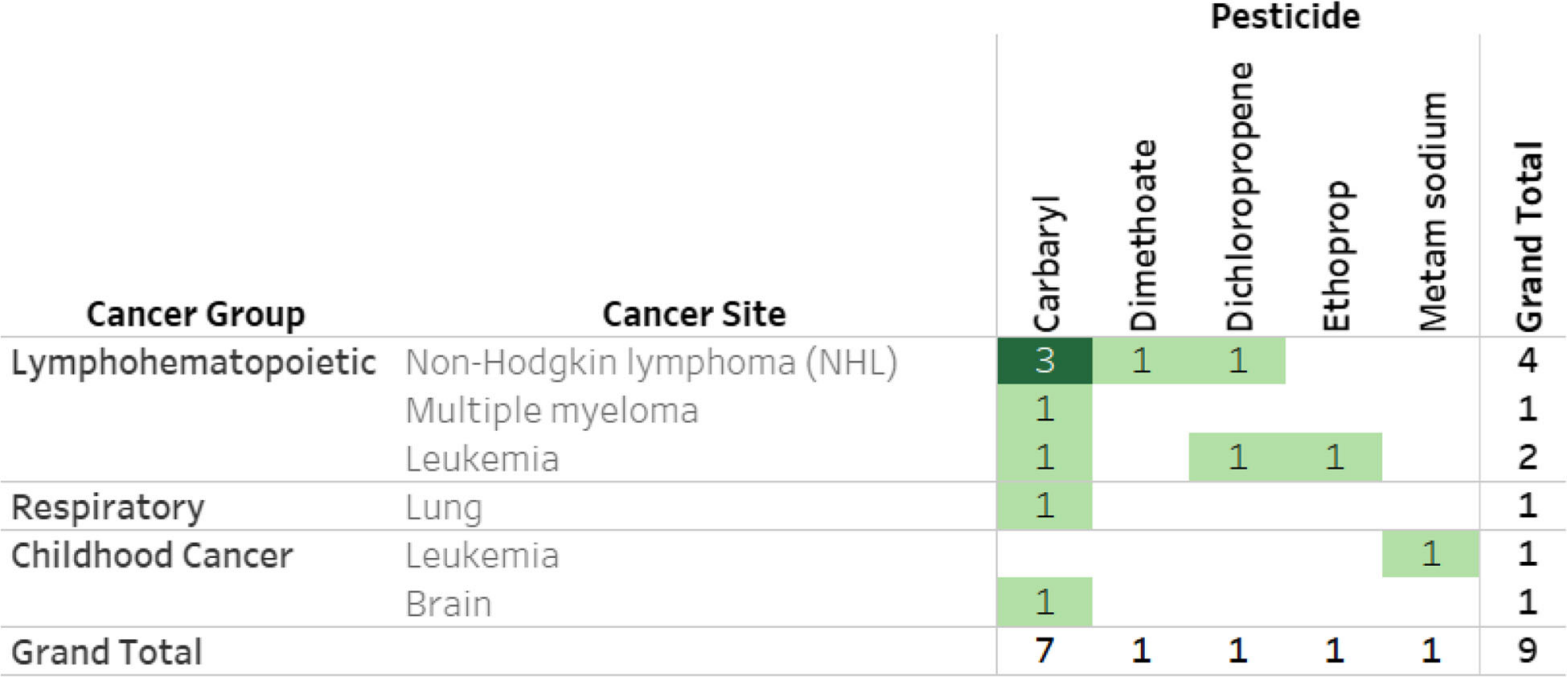
Frequency of cancer epidemiology studies for pesticides with specific risk estimates by cancer site published prior to their carcinogenicity evaluation

Studies for each cell shown online at https://public.tableau.com/profile/ntp.visuals#!/vizhome/ORoCPesticidesCancer081220/ReadMe

## Discussion

### Hazard prioritization and risk assessment

Pesticide registration is a scientifically-based, legal, and administrative process, in which the effects of pesticide use on human health and the environment are assessed [24, 25, 30, 32]. It is also a complex process taking considerable time, resources, and expertise on the part of the registration authority, the pesticide manufacturing industry, and various public interest groups [33]. Given the various guidelines regulating the process and the multiple stakeholders that have an interest in the outcome, USEPA’s process for evaluating carcinogenicity is time-intensive and allows for only a small number of pesticides to be evaluated over time. However, a hazard evaluation conducted when there is a robust database of population-based observational studies published on the carcinogenicity of pesticides can prioritize, inform, and lay the groundwork for a formal risk assessment by USEPA. Preceding a hazard evaluation, this scoping report provides a map of the existing literature on real-world exposures and human cancer that has accumulated on high-volume pesticides classified as potential carcinogens by USEPA, and serves to highlight gaps in the epidemiologic pesticide-human cancer literature.

As noted in the background, there is no standardized approach to prioritizing chemicals for a hazard assessment. Guha et al. [34] identified pesticides by systematically screening chemical structures and creating network maps to visualize clusters of pesticides for chemical similarity, pesticide class, and publicly available information concerning cancer epidemiology, cancer bioassays, and carcinogenic mechanisms. This latter approach yielded a number of agents prioritized for hazard identification including glyphosate, malathion, parathion, tetrachlorvinphos, diazinon, DDT, lindane, and 2,4-dichlorophenoxyacetic acid, all of which have subsequently and recently been reviewed by IARC and found to be probable or possible carcinogens [34]. Compared to the approach outlined in our study, Guha et al. began with chemical similarity network maps for all pesticides. Most of the pesticides they identified with a large number of epidemiologic studies are either not currently registered for use in the U.S., are used in small volumes, or have not been classified as probable or possible carcinogens by USEPA. Additionally, Guha et al. did not specify the number of studies for individual cancers. However, similar to Guha et al., among the pesticides selected by our method, carbaryl, permethrin, metolachlor, and trifluralin had the most epidemiologic studies although the number we found was substantially larger across the pesticides, which is likely due to our method of using full text .pdf searches to find results which is not limited to search titles and abstracts alone.

### Gaps in epidemiologic pesticides and human cancer literature

Key issues for conducting future systematic reviews include the quality of the exposure assessment and potential confounders. As early as 1990, Blair and Zahm [35] noted that improvements in pesticide exposure assessments were necessary if epidemiologic investigations are to provide reliable information on the relationships between cancer incidence and pesticide exposure; however, change has been slow. Ohlander et al. [36] reviewed over 1000 articles on occupational pesticide exposure and a range of health outcomes published between 1993 and 2017, and found that the majority of documented exposure assessment methods were indirect, usually based on self-reported exposure. They also found that the use of self-reported exposures and the specificity of pesticide assessments has increased somewhat over time, primarily due to the decreased used of job titles as proxies. While we identified epidemiologic studies that provide pesticide-specific data for various cancer endpoints, many studies outside of the large agricultural cohorts, in particular those of home and garden exposures, did not provide specific risk estimates for pesticide exposures and/or cancers. Often exposures to pesticides were grouped together in a class (e.g., OPs, carbamates), which has the potential to enable an investigation of common mechanisms by class.

Additional challenges in measuring pesticide exposures include the temporal variability of the use of pesticides across seasons and over time, and the variability in work practices and type of farm operations by country and agricultural commodity. The type of application varies as well, with low-volume pesticides applied via handheld and backpack sprayers potentially leading to far higher exposures than high-volume large-scale field applications. Regarding the specifications of chemicals to which applicators are exposed, there is a lack of knowledge about which chemicals are applied and challenges in ascertaining which formulations of pesticides are used given the ever-evolving changes in formulations over time with the replacement chemicals, and the unknown carcinogenicity of mixtures of parent chemicals with ingredients not necessarily inert. In comparison to the earliest biopersistent organochlorine pesticides, more recent pesticides have shorter biological half-lives precluding the use of exposure biomarkers reflecting long-term exposures typically associated with cancer [37]. More broadly, it is important to recognize the difference in exposure levels of occupational and non-occupational groups, and that studies of high-exposure occupational cohorts are likely to contribute most to our understanding of pesticide-cancer relationships.

Our approach to prioritization could reasonably be extended to currently phased out or “banned” unregistered pesticides which have previously been used in high volumes. Given the long latency of cancers, prioritizing these pesticides would be important for compensation of occupationally related cancers and continued understanding about the impact of these chemicals on health. In addition, investigating banned older chemicals heavily used in the past has value since they tend to be used in low-income countries for a few decades after use ends in higher income countries. Another step may be to search the existing literature on the 290 pesticides we excluded which were classified by USEPA as “not likely to be carcinogenic to humans”, “(having) evidence of non-carcinogenicity in humans”, “not classifiable as a human carcinogen”, or “inadequate data for an assessment of human carcinogenic potential”. For several of these pesticides, there is human population data suggesting carcinogenicity, particularly among some highly-used pesticides (e.g., glyphosate). Chemical structure similarity analysis to the 391 pesticides not evaluated for carcinogenic potential might also be a next step.

Finally, multiple independent study populations are needed to establish consistency for causal inference, and the pesticide-cancer epidemiology literature we identified would benefit from additional studies.

### Next steps

Given that the goal of this study was to identify candidate pesticides for cancer hazard evaluations based on a set of criteria and sufficient epidemiologic literature, we report only the scope of available data, not a formal evaluation of study quality and study results. A hazard evaluation of any particular pesticide would require a systematic evaluation of the quality of each relevant study in relation to a specific cancer site, such as those conducted by NTP. In addition, integration of exposure-disease data across studies, triangulation of these results with other types of study data, integration of relevant data from animal and mechanistic data and a consideration of chemical structure analyses would be undertaken. These steps would lead to informed conclusions about whether the substance should be listed as known or reasonably anticipated to be human carcinogen, based on established criteria [28].

### Study strengths and limitations

The strengths of this analysis are in the use of full-text searching to examine the availability of real-world exposures published in peer-reviewed studies, the use of publicly-available data from USEPA, and data visualization to demonstrate the scope and nature of the evidence in this large database.

We primarily limited our study counts to the most recently published report in a given cohort or population to indicate the scope of the available literature. This may have resulted in de-prioritization of a pesticide, owing to the elimination of earlier studies in a cohort. During the hazard evaluation stage of any chemical, consistency across methods and populations is carefully considered using all studies available. However, due to changes in formulations/mixtures over time, change in use patterns, and differences in biologic responses over time, it is not inconceivable that differing effect estimates can be observed for shorter and longer exposure windows, or in earlier or later analyses. This suggests that during a hazard evaluation, earlier estimates in the same cohort should be considered.

Our findings are limited by available registration and use data. We recognize that these sources are continuously being updated, which may not have been publicly available at the time this report was prepared. For example, as no Federal agency specifically collects information for the purpose of estimating pesticide quantities used on an annual basis by sector, we were limited to using the most recent USEPA report on pesticide use [2] to determine which of those pesticides were used in high volumes in each sector. The latest available data is from 2012, when total U.S. pesticide usage totaled 1.006 billion pounds applied, up from 909 million pounds in 2005 [2]. The USEPA *Sales and Usage* report used estimates compiled from several external sources, including the U.S. Department of Agriculture’s (USDA) National Agriculture Statistics Service (NASS), and from proprietary survey data and research reports of agricultural and non-agricultural use. The 2019 USDA/NASS report on pesticide usage provides more recent data on 430 agricultural pesticides including for the limited period 2015–2017 [38]. Comparing these results with the agricultural sector estimates in the 2016 USEPA report [2], we found that agricultural usage increased from 2012 to 2015–2017 for seven of the 11 agricultural pesticides we identified (Table 2). For the remaining agricultural pesticides, usage was similar in the two reports. More recent data from the state of California are also available [39] but are limited to use in California. In these data, only metam-sodium, pendimethalin, propanil, and mancozeb are among the top 25 pesticides used in the state overall; however, chlorothalonil, trifluralin, metolachlor-S, bifenthrin, dimethoate, and acephate fall within the top 100 most used pesticides. In California, acetochlor is considered a “known” carcinogen and listed on the State of California Proposition 65 Carcinogen List, thus it is no longer in use in California.

## Conclusions

Our scoping study provides a clear picture of human cancer evidence from real-world exposure that has accumulated on high-volume pesticides since their initial carcinogenicity evaluation by USEPA. These results can be used to update authoritative hazard evaluations (e.g., conducted by USEPA, NTP, and/or IARC) on at least eight data-rich pesticides. Our stepwise approach and utilization of evidence mapping can be used to inform future decision-making to update cancer hazard evaluations.

## Supplementary Information

**Additional file 1: Supplemental Table S1.** USEPA carcinogenicity classifications of pesticides over time.

**Additional file 2: Supplemental Table S2.** Reference list of pesticide and cancer epidemiology studies identified in Part 2 of scoping review.

## Data Availability

The data analyzed in the current study are available from USEPA’s Office of Chemical Safety and Pollution Prevention, Office of Pesticide Programs. • USEPA *Pesticide Chemicals Search Database*: https://iaspub.epa.gov/apex/pesticides/f?p=chemicalsearch:1. • USEPA *Chemicals Evaluated for Carcinogenic Potential Annual Cancer Report* [29]*.* • USEPA *Pesticides Industry Sales and Usage, 2008–2012 Market Estimates Report* [2]. Study results are available on a visual dashboard at https://public.tableau.com/profile/ntp.visuals#!/vizhome/ORoCPesticidesCancer081220/ReadMe

## Appendix

### Search terms used to find pesticide references

(“Herbicides”[Mesh] OR Herbicides [tiab] OR Herbicide [tiab] OR “Insecticides”[Mesh] OR Insecticides [tiab] OR Insecticide [tiab] OR “Pesticides”[Mesh] OR Pesticide [tiab] OR Pesticides [tiab] OR “Fungicides Industrial”[Mesh] OR fungicides [tiab] OR fungicide [tiab] OR “Rodenticides”[Mesh] OR Rodenticides [tiab] OR Rodenticide [tiab] OR “Organophosphates”[Mesh] OR Organophosphates [tiab] OR Organophosphate [tiab] OR “Carbamates”[Mesh] OR Carbamates [tiab] OR Carbamate [tiab] OR “Pyrethrins”[Mesh] OR Pyrethrins [tiab] OR Pyrethrin [tiab] OR Pyrethroids [tiab] OR Pyrethroid [tiab] OR Organochlorines [tiab] OR Organochlorine [tiab])

## Abbreviations

ALL: Acute lymphocytic leukemia
DDT: Dichlorodiphenyltrichloroethane
IARC: International Agency for Research on Cancer
HAWC: Health Assessment Workspace Collaborative
NHL: Non-Hodgkin lymphoma
NTP: National Toxicology Program
OP: Organophosphate
OPP: Office of Pesticide Programs
RoC: Report on Carcinogens
USDA/NASS: United States Department of Agriculture / National Agricultural Statistics Service
USEPA: United States Environmental Protection Agency
