# Supplementary figures and images for: A tiered approach to prioritizing registered pesticides for potential cancer hazard evaluations: implications for decision making
Source: Environ Health. 2021 Feb 12;20:13. doi: 10.1186/s12940-021-00696-0 (PMC7881680; doi:10.1186/s12940-021-00696-0)

**
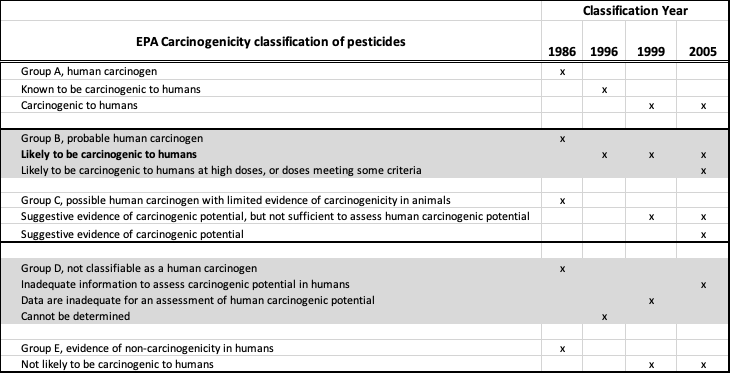
**

Supplement: Supplementary file 1 — Additional file 1: Supplemental Table S1. USEPA carcinogenicity classifications of pesticides over time. [file 12940_2021_696_MOESM1_ESM.docx]
