# Supplementary material for: A tiered approach to prioritizing registered pesticides for potential cancer hazard evaluations: implications for decision making
Source: Environ Health. 2021 Feb 12;20:13. doi: 10.1186/s12940-021-00696-0 (PMC7881680; doi:10.1186/s12940-021-00696-0)
Supplement: Supplementary file 2 — Additional file 2: Supplemental Table S2. Reference list of pesticide and cancer epidemiology studies identified in Part 2 of scoping review. [file 12940_2021_696_MOESM2_ESM.docx]

**Supplemental Table S2.** Reference list of pesticide and cancer epidemiology studies identified in Part 2 of scoping review.

1. Alavanja MC, Hofmann JN, Lynch CF, Hines CJ, Barry KH, Barker J, Buckman DW, Thomas K, Sandler DP, Hoppin JA *et al*: **Non-Hodgkin lymphoma risk and insecticide, fungicide and fumigant use in the Agricultural Health Study**. *PLoS One* 2014, **9**(10):e109332.
2. Alavanja MCR, Coble J, Beane-Freeman LE, Mahajan R, Lubin J, Lynch CF, Knott C, Hoppin JA, Thomas K, Allen R *et al*: **Use of agricultural pesticides and prostate cancer risk in the Agricultural Health Study cohort and future plans for molecular studies**. *Am J Epidemiol* 2006, **163**(11):S113-S113.
3. Alavanja MCR, Dosemeci M, Samanic C, Lubin J, Lynch CF, Knott C, Barker J, Hoppin JA, Sandler DP, Coble J *et al*: **Pesticides and lung cancer risk in the Agricultural Health Study cohort**. *Am J Epidemiol* 2004, **160**(9):876-885.
4. Alavanja MCR, Samanic C, Dosemeci M, Lubin J, Tarone R, Lynch CF, Knott C, Thomas K, Hoppin JA, Barker J *et al*: **Use of agricultural pesticides and prostate cancer risk in the Agricultural Health Study cohort**. *Am J Epidemiol* 2003, **157**(9):800-814.
5. Andreotti G, Freeman LEB, Hou LF, Coble J, Rusiecki J, Hoppin JA, Silverman DT, Alavanja MCR: **Agricultural pesticide use and pancreatic cancer risk in the Agricultural Health Study Cohort**. *Int J Cancer* 2009, **124**(10):2495-2500.
6. Andreotti G, Hou LF, Freeman LEB, Mahajan R, Koutros S, Coble J, Lubin J, Blair A, Hoppin JA, Alavanja M: **Body mass index, agricultural pesticide use, and cancer incidence in the Agricultural Health Study cohort**. *Cancer Causes Control* 2010, **21**(11):1759-1775.
7. Ashley-Martin J, VanLeeuwen J, Cribb A, Andreou P, Guernsey JR: **Breast cancer risk, fungicide exposure and CYP1A1*2A gene-environment interactions in a province-wide case control study in Prince Edward Island, Canada**. *Int J Environ Res Public Health* 2012, **9**(5):1846-1858.
8. Bailey HD, Armstrong BK, de Klerk NH, Fritschi L, Attia J, Scott RJ, Smibert E, Milne E, Aus ALLC: **Exposure to professional pest control treatments and the risk of childhood acute lymphoblastic leukemia**. *Int J Cancer* 2011, **129**(7):1678-1688.
9. Band PR, Abanto Z, Bert J, Lang B, Fang R, Gallagher RP, Le ND: **Prostate cancer risk and exposure to pesticides in British Columbia farmers**. *Prostate* 2011, **71**(2):168-183.
10. Barry KH, Koutros S, Andreotti G, Sandler DP, Burdette LA, Yeager M, Freeman LEB, Lubin JH, Ma XM, Zheng TZ *et al*: **Genetic variation in nucleotide excision repair pathway genes, pesticide exposure and prostate cancer risk**. *Carcinogenesis* 2012, **33**(2):331-337.
11. Barry KH, Koutros S, Bemdt SI, Andreotti G, Hoppin JA, Sandler DP, Burdette LA, Yeager M, Freeman LEB, Lubin JH *et al*: **Genetic variation in base excision repair pathway genes, pesticide exposure, and prostate cancer risk**. *Environ Health Perspect* 2011, **119**(12):1726-1732.
12. Bhat AR, Wani MA, Kirmani AR, Raina TH: **Pesticides and brain cancer linked in orchard farmers of Kashmir**. *Indian J Med Paediatr Oncol* 2010, **31**(4):110-120.
13. Bonner MR, Freeman LEB, Hoppin JA, Koutros S, Sandler DP, Lynch CF, Hines CJ, Thomas K, Blair A, Alavanja MCR: **Occupational exposure to pesticides and the incidence of lung cancer in the Agricultural Health Study**. *Environ Health Perspect* 2017, **125**(4):544-551.
14. Booth BJ, Ward MH, Turyk ME, Stayner LT: **Agricultural crop density and risk of childhood cancer in the midwestern United States: an ecologic study**. *Environmental Health* 2015, **14**.
15. Borkhardt A, Wilda M, Fuchs U, Gortner L, Reiss I: **Congenital leukaemia after heavy abuse of permethrin during pregnancy**. *Arch Dis Child Fetal Neonatal Ed* 2003, **88**(5):F436-F437.
16. Boulanger M, Tual S, Lemarchand C, Guizard AV, Delafosse P, Marcotullio E, Pons R, Piel C, Pouchieu C, Baldi I *et al*: **Lung cancer risk and occupational exposures in crop farming: results from the AGRIculture and CANcer (AGRICAN) cohort**. *Occup Environ Med* 2018, **75**(11):776-785.
17. Brody JG, Aschengrau A, McKelvey W, Rudel RA, Swartz CH, Kennedy T: **Breast cancer risk and historical exposure to pesticides from wide-area applications assessed with GIS**. *Environ Health Perspect* 2004, **112**(8):889-897.
18. Brown LM, Blair A, Gibson R, Everett GD, Cantor KP, Schuman LM, Burmeister LF, Van Lier SF, Dick F: **Pesticide exposures and other agricultural risk factors for leukemia among men in Iowa and Minnesota**. *Cancer Res* 1990, **50**(20):6585-6591.
19. Brown LM, Burmeister LF, Everett GD, Blair A: **Pesticide exposures and multiple-myeloma in Iowa men**. *Cancer Causes Control* 1993, **4**(2):153-156.
20. Cantor KP, Blair A, Brown LM, Burmeister LF, Everett G: **Correspondence re: K. P. Cantor et al., pesticides and other agricultural risk factors for non-Hodgkin's lymphoma among men in Iowa and Minnesota, Cancer Res., 52: 2447-2455, 1992**. *Cancer Res* 1993, **53**(10 Suppl):2421.
21. Cancer Facts and Figures, 2020. American Cancer Society. https://www.cancer.org/content/dam/cancer-org/research/cancer-facts-and-statistics/annual-cancer-facts-and-figures/2020/cancer-facts-and-figures-2020.pdf
22. Cantor KP, Blair A, Everett G, Gibson R, Burmeister LF, Brown LM, Schuman L, Dick FR: **Pesticides and other agricultural risk factors for non-Hodgkin's lymphoma among men in Iowa and Minnesota**. *Cancer Res* 1992, **52**(9):2447-2455.
23. Carozza SE, Li B, Elgethun K, Whitworth R: **Risk of childhood cancers associated with residence in agriculturally intense areas in the United States**. *Environ Health Perspect* 2008, **116**(4):559-565.
24. Carreon T, Butler MA, Ruder AM, Waters MA, Davis-King KE, Calvert GM, Schulte PA, Connally B, Ward EM, Sanderson WT *et al*: **Gliomas and farm pesticide exposure in women: The Upper Midwest Health Study**. *Environ Health Perspect* 2005, **113**(5):546-551.
25. Cha ES, Hwang SS, Lee WJ: **Childhood leukemia mortality and farming exposure in South Korea: A national population-based birth cohort study**. *Cancer Epidemiol* 2014, **38**(4):401-407.
26. Chatterjee SK, Forson SAK, Kalojanova F: **Recommended Health-Based Limits in Occupational Exposure to Pesticides**. Technical Report Series No. 677. Geneva: World Health Organization; 1982. https://apps.who.int/iris/handle/10665/41535
27. Christensen CH, Barry KH, Andreotti G, Alavanja MCR, Cook MB, Kelly SP, Burdett LA, Yeager M, Freeman LEB, Berndt SI *et al*: **Sex steroid hormone single-nucleotide polymorphisms, pesticide use, and the risk of prostate cancer: A nested case-control study within the Agricultural Health Study**. *Front Oncol* 2016, **6**.
28. Cocco P, Satta G, Dubois S, Pili C, Pilleri M, Zucca M, t'Mannetje AM, Becker N, Benavente Y, de Sanjose S *et al*: **Lymphoma risk and occupational exposure to pesticides: results of the Epilymph Study**. *Occup Environ Med* 2013, **70**(2):91-98.
29. Colli JL, Kolettis PN: **Bladder cancer incidence and mortality rates compared to ecologic factors among states in America**. *Int Urol Nephrol* 2010, **42**(3):659-665.
30. Colt JS, Davis S, Severson RK, Lynch CF, Cozen W, Camann D, Engels EA, Blair A, Hartge P: **Residential insecticide use and risk of non-Hodgkin's lymphoma**. *Cancer Epidemiol Biomarkers Prev* 2006, **15**(2):251-257.
31. Cooney MA, Daniels JL, Ross JA, Breslow NE, Pollock BH, Olshan AF: **Household pesticides and the risk of Wilms tumor**. *Environ Health Perspect* 2007, **115**(1):134-137.
32. Czarnota J, Gennings C, Colt JS, De Roos AJ, Cerhan JR, Severson RK, Hartge P, Ward MH, Wheeler DC: **Analysis of environmental chemical mixtures and non-Hodgkin lymphoma risk in the NCI-SEER NHL Study**. *Environ Health Perspect* 2015, **123**(10):965-970.
33. Davis JR, Brownson RC, Garcia R, Bentz BJ, Turner A: **Family pesticide use and childhood brain cancer**. *Arch Environ Contam Toxicol* 1993, **24**(1):87-92.
34. De Roos AJ, Blair A, Rusiecki JA, Hoppin JA, Svec M, Dosemeci M, Sandler DP, Alavanja MC: **Cancer incidence among glyphosate-exposed pesticide applicators in the agricultural health study**. *Environ Health Perspect* 2005, **113**(1):49-54.
35. De Roos AJ, Zahm SH, Cantor KP, Weisenburger DD, Holmes FF, Burmeister LF, Blair A: **Integrative assessment of multiple pesticides as risk factors for non-Hodgkin's lymphoma among men**. *Occup Environ Med* 2003, **60**(9).
36. Dennis LK, Lynch CF, Sandler DP, Alavanja MCR: **Pesticide use and cutaneous melanoma in pesticide applicators in the Agricultural Heath Study**. *Environ Health Perspect* 2010, **118**(6):812-817.
37. Ding GD, Shi R, Gao Y, Zhang Y, Kamijima M, Sakai K, Wang GQ, Feng C, Tian Y: **Pyrethroid pesticide exposure and risk of childhood acute lymphocytic leukemia in Shanghai**. *Environ Sci Technol* 2012, **46**(24):13480-13487.
38. El-Zaemey S, Heyworth J, Fritschi L: **Noticing pesticide spray drift from agricultural pesticide application areas and breast cancer: a case-control study**. *Aust NZ J Public Health* 2013, **37**(6):547-555.
39. Engel LS, Hill DA, Hoppin JA, Lubin JH, Lynch CF, Pierce J, Samanic C, Sandler DP, Blair A, Alavanja MC: **Pesticide use and breast cancer risk among farmers' wives in the agricultural health study**. *Am J Epidemiol* 2005, **161**(2):121-135.
40. Ferreira JD, Couto AC, Pombo-de-Oliveira MS, Koifman S, Brazilian Collaborative Study G: ***In utero* pesticide exposure and leukemia in Brazilian children < 2 years of age**. *Environ Health Perspect* 2013, **121**(2):269-275.
41. Flower KB, Hoppin JA, Lynch CF, Blair A, Knott C, Shore DL, Sandler DP: **Cancer risk and parental pesticide application in children's of Agricultural Health Study participants**. *Environ Health Perspect* 2004, **112**(5):631-635.
42. Fortes C, Mastroeni S, Segatto MM, Hohmann C, Miligi L, Bakos L, Bonamigo R: **Occupational exposure to pesticides with occupational sun exposure increases the risk for cutaneous melanoma**. *J Occup Environ Med* 2016, **58**(4):370-375.
43. Gambini GF, Mantovani C, Pira E, Piolatto PG, Negri E: **Cancer mortality among rice growers in Novara Province, Northern Italy**. *Am J Ind Med* 1997, **31**(4):435-441.
44. Greenop KR, Peters S, Bailey HD, Fritschi L, Attia J, Scott RJ, Glass DC, de Klerk NH, Alvaro F, Armstrong BK *et al*: **Exposure to pesticides and the risk of childhood brain tumors**. *Cancer Causes Control* 2013, **24**(7):1269-1278.
45. Hoar SK, Blair A, Holmes FF, Boysen CD, Robel RJ, Hoover R, Fraumeni JF, Jr.: **Agricultural herbicide use and risk of lymphoma and soft-tissue sarcoma**. *JAMA* 1986, **256**(9):1141-1147.
46. Hohenadel K, Harris SA, McLaughlin JR, Spinelli JJ, Pahwa P, Dosman JA, Demers PA, Blair A: **Exposure to multiple pesticides and risk of non-Hodgkin lymphoma in men from six Canadian provinces**. *Int J Environ Res Public Health* 2011, **8**(6):2320-2330.
47. Hou LF, Lee WJ, Rusiecki J, Hoppin JA, Blair A, Bonner MR, Lubin JH, Samanic C, Sandler DP, Dosemeci M *et al*: **Pendimethalin exposure and cancer incidence among pesticide applicators**. *Epidemiology* 2006, **17**(3):302-307.
48. Hyland C, Gunier RB, Metayer C, Bates MN, Wesseling C, Mora AM: **Maternal residential pesticide use and risk of childhood leukemia in Costa Rica**. *Int J Cancer* 2018, **143**(6):1295-1304.
49. Jones RR, Yu CL, Nuckols JR, Cerhan JR, Airola M, Ross JA, Robien K, Ward MH: **Farm residence and lymphohematopoietic cancers in the Iowa Women's Health Study**. *Environmental Research* 2014, **133**:353-361.
50. Kachuri L, Demers PA, Blair A, Spinelli JJ, Pahwa M, McLaughlin JR, Pahwa P, Dosman JA, Harris SA: **Multiple pesticide exposures and the risk of multiple myeloma in Canadian men**. *Int J Cancer* 2013, **133**(8):1846-1858.
51. Kang D, Park SK, Beane-Freeman L, Lynch CF, Knott CE, Sandler DP, Hoppin JA, Dosemeci M, Coble J, Lubin J *et al*: **Cancer incidence among pesticide applicators exposed to trifluralin in the Agricultural Health Study**. *Environ Res* 2008, **107**(2):271-276.
52. Karami S, Andreotti G, Koutros S, Barry KH, Moore LE, Han S, Hoppin JA, Sandler DP, Lubin JH, Burdette LA *et al*: **Pesticide Exposure and Inherited Variants in Vitamin D Pathway Genes in Relation to Prostate Cancer**. *Cancer Epidemiol Biomarkers Prev* 2013, **22**(9):1557-1566.
53. Kaufman DW, Anderson TE, Issaragrisil S: **Risk factors for leukemia in Thailand**. *Ann Hematol* 2009, **88**(11):1079-1088.
54. Kokouva M, Bitsolas N, Hadjigeorgiou GM, Rachiotis G, Papadoulis N, Hadjichristodoulou C: **Pesticide exposure and lymphohaematopoietic cancers: a case-control study in an agricultural region (Larissa, Thessaly, Greece)**. *BMC Public Health* 2011, **11**.
55. Koutros S, Andreotti G, Berndt SI, Hughes Barry K, Lubin JH, Hoppin JA, Kamel F, Sandler DP, Burdette LA, Yuenger J *et al*: **Xenobiotic-metabolizing gene variants, pesticide use, and the risk of prostate cancer**. *Pharmacogenet Genom* 2011, **21**(10):615-623.
56. Koutros S, Beane Freeman LE, Berndt SI, Andreotti G, Lubin JH, Sandler DP, Hoppin JA, Yu K, Li Q, Burdette LA *et al*: **Pesticide use modifies the association between genetic variants on chromosome 8q24 and prostate cancer**. *Cancer Res* 2010, **70**(22):9224-9233.
57. Koutros S, Silverman DT, Alavanja MCR, Andreotti G, Lerro CC, Heltshe S, Lynch CF, Sandler DP, Blair A, Freeman LEB: **Occupational exposure to pesticides and bladder cancer risk**. *Int J Epidemiol* 2016, **45**(3):792-805.
58. Landgren O, Kyle RA, Hoppin JA, Freeman LEB, Cerhan JR, Katzmann JA, Rajkumar SV, Alavanja MC: **Pesticide exposure and risk of monoclonal gammopathy of undetermined significance in the Agricultural Health Study**. *Blood* 2009, **113**(25):6386-6391.
59. Lee WJ, Cantor KP, Berzofsky JA, Zahn SH, Blair A: **Non-Hodgkin's lymphoma among asthmatics exposed to pesticides**. *Int J Cancer* 2004, **111**(2):298-302.
60. Lee WJ, Colt JS, Heineman EF, McComb R, Weisenburger DD, Lijinsky W, Ward MH: **Agricultural pesticide use and risk of glioma in Nebraska, United States**. *Occup Environ Med* 2005, **62**(11).
61. Lee WJ, Lijinsky W, Heineman EF, Markin RS, Weisenburger DD, Ward MH: **Agricultural pesticide use and adenocarcinomas of the stomach and oesophagus**. *Occup Environ Med* 2004, **61**(9):743-749.
62. Lee WJ, Sandler DP, Blair A, Samanic C, Cross AJ, Alavanja MCR: **Pesticide use and colorectal cancer risk in the Agricultural Health Study**. *Int J Cancer* 2007, **121**(2):339-346.
63. Leiss JK, Savitz DA: **Home pesticide use and childhood cancer: a case-control study**. *Am J Public Health* 1995, **85**(2):249-252.
64. Leon ME, Schinasi LH, Lebailly P, Beane Freeman LE, Nordby KC, Ferro G, Monnereau A, Brouwer M, Tual S, Baldi I *et al*: **Pesticide use and risk of non-Hodgkin lymphoid malignancies in agricultural cohorts from France, Norway and the USA: a pooled analysis from the AGRICOH consortium**. *Int J Epidemiol* 2019.
65. Lerro CC, Koutros S, Andreotti G, Hines CJ, Blair A, Lubin J, Ma XM, Zhang YW, Beane Freeman LE: **Use of acetochlor and cancer incidence in the Agricultural Health Study**. *Int J Cancer* 2015, **137**(5):1167-1175.
66. Littorin M, Attewell R, Skerfving S, Horstmann V, Moller T: **Mortality and tumor morbidity among Swedish market gardeners and orchardists**. *Int Arch Occup Environ Health* 1993, **65**(3):163-169.
67. Mahajan R, Blair A, Coble J, Lynch CF, Hoppin JA, Sandler DP, Alavanja MCR: **Carbaryl exposure and incident cancer in the Agricultural Health Study**. *Int J Cancer* 2007, **121**(8):1799-1805.
68. Malagoli C, Costanzini S, Heck JE, Malavolti M, De Girolamo G, Oleari P, Palazzi G, Teggi S, Vinceti M: **Passive exposure to agricultural pesticides and risk of childhood leukemia in an Italian community**. *Int J Hyg Environ Health* 2016, **219**(8):742-748.
69. McDuffie HH, Pahwa P, McLaughlin JR, Spinelli JJ, Fincham S, Dosman JA, Robson D, Skinnider LF, Choi NW: **Non-Hodgkin's lymphoma and specific pesticide exposures in men: cross-Canada study of pesticides and health**. *Cancer Epidemiol Biomarkers Prev* 2001, **10**(11):1155-1163.
70. Menegaux F, Baruchel A, Bertrand Y, Lescoeur B, Leverger G, Nelken B, Sammelet D, Hémon D, Clavel J: **Household exposure to pesticides and risk of childhood acute leukaemia**. *Occup Environ Med* 2006, **63**(2):131-134.
71. Metayer C, Colt JS, Buffler PA, Reed HD, Selvin S, Crouse V, Ward MH: **Exposure to herbicides in house dust and risk of childhood acute lymphoblastic leukemia**. *J Expo Sci Environ Epidemiol* 2013, **23**(4):363-370.
72. Miligi L, Costantini AS, Bolejack V, Veraldi A, Benvenuti A, Nanni O, Ramazzotti V, Tumino R, Stagnaro E, Rodella S *et al*: **Non-Hodgkin's lymphoma, leukemia, and exposures in agriculture: Results from the Italian multicenter case-control study**. *Am J Ind Med* 2003, **44**(6):627-636.
73. Miligi L, Settimi L, Masala G, Maiozzi P, Alberghini Maltoni S, Seniori Costantini A, Vineis P: **Pesticide exposure assessment: A crop exposure matrix**. *Int J Epidemiol* 1993, **22**:S42-S45.
74. Mills PK: **Correlation analysis of pesticide use data and cancer incidence rates in California counties**. *Arch Environ Health* 1998, **53**(6):410-413.
75. Mills PK, Shah P: **Cancer incidence in California farm workers, 1988-2010**. *Am J Ind Med* 2014, **57**(7):737-747.
76. Mills PK, Yang R, Riordan D: **Lymphohematopoietic cancers in the United Farm Workers of America (UFW), 1988-2001**. *Cancer Causes Control* 2005, **16**(7):823-830.
77. Mills PK, Yang RC: **Agricultural exposures and gastric cancer risk in Hispanic farm workers in California**. *Environ Res* 2007, **104**(2):282-289.
78. Mozzachio AM, Rusiecki JA, Hoppin JA, Mahajan R, Patel R, Beane-Freeman L, Alavanja MCR: **Chlorothalonil exposure and cancer incidence among pesticide applicator participants in the Agricultural Health Study**. *Environmental Research* 2008, **108**(3):400-403.
79. Nanni O, Amadori D, Lugaresi C, Falcini F, Scarpi E, Saragoni A, Buiatti E: **Chronic lymphocytic leukaemias and non-Hodgkin's lymphomas by histological type in farming-animal breeding workers: A population case-control study based on a priori exposure matrices**. *Occup Environ Med* 1996, **53**(10):652-657.
80. Navaranjan G, Hohenadel K, Blair A, Demers PA, Spinelli JJ, Pahwa P, McLaughlin JR, Dosman JA, Ritter L, Harris SA: **Exposures to multiple pesticides and the risk of Hodgkin lymphoma in Canadian men**. *Cancer Causes Control* 2013, **24**(9):1661-1673.
81. Nicolle-Mir L: **Exposure to pesticides and lung cancer in the Agricultural Health Study**. *Environ Risques Sante* 2017, **16**:544-551.
82. Nordby KC, Andersen A, Irgens LM, Kristensen P: **Indicators of mancozeb exposure in relation to thyroid cancer and neural tube defects in farmers' families**. *Scand J Work Environ Health* 2005, **31**(2):89-96.
83. Omidakhsh N, Ganguly A, Bunin GR, von Ehrenstein OS, Ritz B, Heck JE: **Residential pesticide exposures in pregnancy and the risk of sporadic retinoblastoma: A report from the Children's Oncology Group**. *Am J Ophthalmol* 2017, **176**:166-173.
84. Pahwa M, Harris SA, Hohenadel K, McLaughlin JR, Spinelli JJ, Pahwa P, Dosman JA, Blair A: **Pesticide use, immunologic conditions, and risk of non-Hodgkin lymphoma in Canadian men in six provinces**. *Int J Cancer* 2012, **131**(11):2650-2659.
85. Pahwa P, Karunanayake CP, Dosman JA, Spinelli JJ, McDuffie HH, McLaughlin JR: **Multiple myeloma and exposure to pesticides: a Canadian case-control study**. *J Agromedicine* 2012, **17**(1):40-50.
86. Pesatori AC, Sontag JM, Lubin JH, Consonni D, Blair A: **Cohort mortality and nested case-control study of lung-cancer among structural pest-control workers in Florida (United States)**. *Cancer Causes Control* 1994, **5**(4):310-318.
87. Piel C, Pouchieu C, Migault L, Beziat B, Boulanger M, Bureau M, Carles C, Gruber A, Lecluse Y, Rondeau V *et al*: **Increased risk of central nervous system tumours with carbamate insecticide use in the prospective cohort AGRICAN**. *Int J Epidemiol* 2018.
88. Pimentel D, Burgess M: **Environmental and economic costs of the application of pesticides primarily in the United States**. In: *Integrated Pest Management: Pesticide Problems.* Edited by Pimentel D, Peshin R, vol. 3. Dordrecht: Springer Science+Business Media; 2013: pp. 47-71.
89. Pogoda JM, Preston-Martin S: **Household pesticides and risk of pediatric brain tumors**. *Environ Health Perspect* 1997, **105**(11):1214-1220.
90. Presutti R, Harris SA, Kachuri L, Spinelli JJ, Pahwa M, Blair A, Zahm SH, Cantor KP, Weisenburger DD, Pahwa P *et al*: **Pesticide exposures and the risk of multiple myeloma in men: An analysis of the North American Pooled Project**. *Int J Cancer* 2016, **139**(8):1703-1714.
91. Provost D, Cantagrel A, Lebailly P, Jaffre A, Loyant V, Loiseau H, Vital A, Brochard P, Baldi I: **Brain tumours and exposure to pesticides: a case-control study in southwestern France**. *Occup Environ Med* 2007, **64**(8):509-514.
92. Reynolds P, Hurley SE, Gunier RB, Yerabati S, Quach T, Hertz A: **Residential proximity to agricultural pesticide use and incidence of breast cancer in California, 1988-1997**. *Environ Health Perspect* 2005, **113**(8):993-1000.
93. Reynolds P, Von Behren J, Gunier RB, Goldberg DE, Harnly M, Hertz A: **Agricultural pesticide use and childhood cancer in California**. *Epidemiology* 2005, **16**(1):93-100.
94. Reynolds P, Von Behren J, Gunier RB, Goldberg DE, Hertz A, Harnly ME: **Childhood cancer and agricultural pesticide use: an ecologic study in California**. *Environ Health Perspect* 2002, **110**(3):319-324.
95. Ruder AM, Waters MA, Butler MA, Carreon T, Calvert GM, Davis-King KE, Schulte PA, Sanderson WT, Ward EM, Connally LB *et al*: **Gliomas and farm pesticide exposure in men: The upper midwest health study**. *Arch Environ Health* 2004, **59**(12):650-657.
96. Rull RP, Gunier R, Von Behren J, Hertz A, Crouse V, Buffler PA, Reynolds P: **Residential proximity to agricultural pesticide applications and childhood acute lymphoblastic leukemia**. *Environmental Research* 2009, **109**(7):891-899.
97. Rusiecki JA, Hou LF, Lee WJ, Blair A, Dosemeci M, Lubin JH, Bonner M, Samanic C, Hoppin JA, Sandler DP *et al*: **Cancer incidence among pesticide applicators exposed to metolachlor in the Agricultural Health Study**. *Int J Cancer* 2006, **118**(12):3118-3123.
98. Rusiecki JA, Patel R, Koutros S, Beane-Freeman L, Landgren O, Bonner MR, Coble J, Lubin J, Blair A, Hoppin JA *et al*: **Cancer incidence among pesticide applicators exposed to permethrin in the Agricultural Health Study**. *Environ Health Perspect* 2009, **117**(4):581-586.
99. Salerno C, Carcagni A, Sacco S, Palin LA, Vanhaecht K, Panella M, Guido D: **An Italian population-based case-control study on the association between farming and cancer: Are pesticides a plausible risk factor?** *Arch Environ Occup Health* 2016, **71**(3):147-156.
100. Schinasi L, Leon ME: **Non-hodgkin lymphoma and occupational exposure to agricultural pesticide chemical groups and active ingredients: A systematic review and meta-analysis**. *Int J Environ Res Public Health* 2014, **11**(4):4449-4527.
101. Schinasi LH, De Roos AJ, Ray RM, Edlefsen KL, Parks CG, Howard BV, Meliker JR, Bonner MR, Wallace RB, LaCroix AZ: **Insecticide exposure and farm history in relation to risk of lymphomas and leukemias in the Women's Health Initiative observational study cohort**. *Ann Epidemiol* 2015, **25**(11):803-810.
102. Schreinemachers DM, Creason JP, Garry VF: **Cancer mortality in agricultural regions of Minnesota**. *Environ Health Perspect* 1999, **107**(3):205-211.
103. Segatto MM, Bonamigo RR, Hohmann CB, Muller KR, Bakos L, Mastroeni S, Fortes C: **Residential and occupational exposure to pesticides may increase risk for cutaneous melanoma: a case-control study conducted in the south of Brazil**. *Int J Dermatol* 2015, **54**(12):E527-E538.
104. Shim YK, Mlynarek SP, van Wijngaarden E: **Parental exposure to pesticides and childhood brain cancer: US Atlantic Coast Childhood Brain Cancer Study**. *Environ Health Perspect* 2009, **117**(6):1002-1006.
105. Silver SR, Bertke SJ, Hines CJ, Alavanja MCR, Hoppin JA, Lubin JH, Rusiecki JA, Sandler DP, Freeman LEB: **Cancer incidence and metolachlor use in the Agricultural Health Study: An update**. *Int J Cancer* 2015, **137**(11):2630-2643.
106. Soldin OP, Nsouly-Maktabi H, Genkinger JM, Loffredo CA, Ortega-Garcia JA, Colantino D, Barr DB, Luban NL, Shad AT, Nelson D: **Pediatric acute lymphoblastic leukemia and exposure to pesticides**. *Therap Drug Monitor* 2009, **31**(4):495-501.
107. Sorahan T: **Multiple myeloma and glyphosate use: a re-analysis of US Agricultural Health Study (AHS) data**. *Int J Environ Res Public Health* 2015, **12**(2):1548-1559.
108. Teitelbaum SL, Gammon MD, Britton JA, Neugut AI, Levin B, Stellman SD: **Reported residential pesticide use and breast cancer risk on Long Island, New York**. *Am J Epidemiol* 2007, **165**(6):643-651.
109. Thomas HF, Winter PD, Donaldson LJ: **Cancer mortality among local authority pest control officers in England and Wales**. *Occup Environ Med* 1996, **53**(11):787-790.
110. Turner MC, Wigle DT, Krewski D: **Residential pesticides and childhood leukemia: a systematic review and meta-analysis**. *Ciencia & Saude Coletiva* 2011, **16**(3):1915-1931.
111. Van Maele-Fabry G, Willems JL: **Prostate cancer among pesticide applicators: a meta-analysis**. *Int Arch Occup Environ Health* 2004, **77**(8):559-570.
112. Waddell BL, Zahm SH, Baris D, Weisenburger DD, Holmes F, Burmeister LF, Cantor KP, Blair A: **Agricultural use of organophosphate pesticides and the risk of non-Hodgkin's lymphoma among male farmers (United States)**. *Cancer Causes Control* 2001, **12**(6):509-517.
113. Wesseling C, Ahlbom A, Antich D, Rodriguez AC, Castro R: **Cancer in banana plantation workers in Costa Rica**. *Int J Epidemiol* 1996, **25**(6):1125-1131.
114. Wesseling C, Antich D, Hogstedt C, Rodriguez AC, Ahlbom A: **Geographical differences of cancer incidence in Costa Rica in relation to environmental and occupational pesticide exposure**. *Int J Epidemiol* 1999, **28**(3):365-374.
115. Yiin JH, Ruder AM, Stewart PA, Waters MA, Carreon T, Butler MA, Calvert GM, Davis-King KE, Schulte PA, Mandel JS *et al*: **The upper midwest health study: a case-control study of pesticide applicators and risk of glioma**. *Environmental Health* 2012, **11**.
116. Zahm SH, Weisenburger DD, Saal RC, Vaught JB, Babbitt PA, Blair A: **The role of agricultural pesticide use in the development of non-Hodgkin's lymphoma in women**. *Arch Environ Health* 1993, **48**(5):353-358.
117. Zheng T, Zahm SH, Cantor KP, Weisenburger DD, Zhang Y, Blair A: **Agricultural exposure to carbamate pesticides and risk of non-Hodgkin lymphoma**. *J Occup Environ Med* 2001, **43**(7):641-649.
118. Zota AR, Aschengrau A, Rudel RA, Brody JG: **Self-reported chemicals exposure, beliefs about disease causation, and risk of breast cancer in the Cape Cod Breast Cancer and Environment Study: a case-control study**. *Environmental Health* 2010, **9**.
